# FAERS-based pharmacovigilance and network toxicology analysis of esomeprazole-associated osteoporosis

**DOI:** 10.1371/journal.pone.0353264

**Published:** 2026-08-04

**Authors:** Yan Wang, Xiaolong Xu, Guanjun Huang, Hongyan Zhu, Xuan Li, Jue Lin

**Affiliations:** 1 Department of Endocrinology, Shangrao Hospital affiliated to Nanchang University, Shangrao, Jiangxi, China; 2 Department of Gastroenterology, Shangrao Hospital affiliated to Nanchang University, Shangrao, Jiangxi, China; Icahn School of Medicine at Mount Sinai Department of Pharmacological Sciences, UNITED STATES OF AMERICA

## Abstract

**Background:**

Osteoporosis (OP) is a progressive skeletal disorder often exacerbated by pharmacological agents. Proton pump inhibitors (PPIs), particularly esomeprazole, have been epidemiologically linked to OP, but the underlying mechanisms remain unclear.

**Methods:**

This study mined over 55 million adverse event (AE) records from the FDA Adverse Event Reporting System (FAERS) between 2004 and 2024 to identify drug-related OP signals. Four statistical algorithms (ROR, PRR, BCPNN, EBGM) were applied for signal detection. Logistic regression was used to assess independent risk factors. Esomeprazole, the most frequently reported PPI, was further investigated using a network toxicology framework. Drug-target predictions, differential gene expression analysis, protein-protein interaction network construction, molecular docking and molecular dynamics (MD) simulations were conducted to explore potential molecular hypotheses.

**Results:**

A total of 52 drugs showed potential OP-related reporting signals. Among PPI-associated OP-related reports, esomeprazole accounted for the largest proportion and was the only PPI with positive signals across all four algorithms. Logistic regression showed that esomeprazole, age, sex, and low body weight were associated with OP-related adverse event reporting. Time-to-onset analysis showed a median latency of 3.5 years. Network toxicology analysis identified 26 overlapping candidate genes between predicted esomeprazole targets and OP-related DEGs. GO and KEGG analyses highlighted bone-related pathways, including MAPK, Notch, and PI3K/AKT signaling. Protein-protein interaction network analysis identified CXCR4, AKT1, and CASP3 as candidate hub genes. Molecular docking and 100-ns MD simulations supported the binding plausibility and conformational stability of the predicted esomeprazole-target complexes.

**Conclusion:**

This pharmacovigilance and computational toxicology study suggests a notable association between esomeprazole and PPI-associated OP-related adverse event reporting. The identified targets and pathways, including CXCR4, AKT1, and CASP3, provide exploratory mechanistic clues and hypothesis-generating evidence. These findings may support individualized bone health risk assessment in long-term PPI users and provide a basis for future validation studies.

## 1. Introduction

Osteoporosis (OP) is a systemic skeletal disorder characterized by reduced bone mass and deterioration of trabecular microarchitecture, leading to increased bone fragility and a heightened risk of fractures [1,2]. According to the World Health Organization (WHO), OP is defined as a bone mineral density (BMD) that is 2.5 standard deviations or more below the average for a healthy young adult [3]. In recent years, the global prevalence of OP has continued to rise, with over 200 million individuals currently affected worldwide [4]. Osteoporotic fractures not only severely impair patients’ quality of life but also impose a substantial economic burden [5]. Therefore, identifying and mitigating risk factors for OP is of great public health importance to reduce fracture incidence and alleviate the socioeconomic impact.

In addition to well-established risk factors such as aging, lifestyle, and genetic predisposition, pharmacologic agents have emerged as key contributors to OP [1]. In recent years, drug-induced OP has garnered growing attention. A variety of commonly prescribed medications—including glucocorticoids and PPIs—have been shown to interfere with bone metabolism through diverse mechanisms, ultimately increasing the risk of bone loss and fractures [6,7].

Among these, PPIs are of particular concern due to their widespread use in the treatment of acid-related gastrointestinal disorders. Epidemiological studies have reported a significant association between PPI use and increased risks of hip and vertebral fractures, with a potential dose- and duration-dependent relationship [8,9]. Although this has traditionally been attributed to impaired calcium absorption due to gastric acid suppression, accumulating evidence suggests that calcium supplementation alone does not fully reverse the decline in BMD observed in PPI users [8,10], implying the involvement of additional, more complex mechanisms. Moreover, the effects of individual PPI agents on bone health remain controversial. Some studies suggest that esomeprazole has no significant impact on bone homeostasis, while others report a notable association with reduced bone mineral density [11–13], highlighting the need for further mechanistic investigation.

To systematically explore the relationship between PPIs and OP, we employed data from the U.S. Food and Drug Administration’s Adverse Event Reporting System to investigate drug-related OP cases reported between 2004 and 2024. As the largest spontaneous adverse event reporting system globally, FAERS is widely used for pharmacovigilance and signal detection [14]. By applying multiple statistical algorithms—including the ROR, PRR, BCPNN, and EBGM—we quantified the strength of associations between drugs and OP-related adverse events to identify high-risk medications.

In total, we identified 52 drugs potentially associated with OP. Among them, PPIs were frequently reported, with esomeprazole emerging as the most prominent. It accounted for 87.6% of PPI-related OP reports and was the only PPIs to exhibit positive signals across all four detection algorithms, suggesting a robust and significant association with OP.

Based on these findings, we further investigated potential molecular links between esomeprazole and OP-related biological processes. By integrating compound-target prediction, public transcriptomic datasets, differential gene expression analysis, protein-protein interaction network construction, and molecular docking, we systematically explored the key regulatory pathways and candidate targets involved from a network toxicology perspective. These findings provide exploratory evidence for understanding potential PPI-associated bone effects and may support more individualized risk assessment in clinical practice. This study adheres to the TITAN 2025 guidelines for transparency in the reporting of artificial intelligence use [15].

## 2. Materials and methods

### 2.1 Data extraction and preprocessing

All data were obtained from the U.S. Food and Drug Administration (FDA) Adverse Event Reporting System(FAERS), which comprises seven relational datasets: Demographics (DEMO), Drug Information (DRUG), Indications (INDI), Adverse Events (REAC), Outcomes (OUTC), Report Sources (RPSR), and Therapy Dates (THER). FAERS is updated and released quarterly.

We retrieved and analyzed all AE reports related to OP from Q1 2004 to Q4 2024. Data de-duplication was performed following FDA recommendations, using CASEID as the primary key: when multiple entries existed for the same case, the record with the latest FDA_DT was retained; if FDA_DT was identical, the one with the higher PRIMARYID was preserved [16].

Adverse events were standardized using Preferred Terms (PTs) from the Medical Dictionary for Regulatory Activities (MedDRA) version 26.1. OP-related AEs included “osteoporosis” and “Osteoporotic fracture.” Only reports listing the study drug as the primary suspect drug were included. Key extracted variables included patient age, sex, reporting country, drug name, and indications.

### 2.2 Signal detection of AEs

Four established signal detection algorithms were used to evaluate the potential associations between PPIs and OP: reporting odds ratio (ROR), proportional reporting ratio (PRR), Bayesian confidence propagation neural network (BCPNN), and empirical Bayes geometric mean (EBGM). All methods were based on 2 × 2 contingency tables (see S1 Table), and their formulas and statistical thresholds are provided in S2 Table.

### 2.3 Time-to-onset analysis

Time to onset (TTO) of AEs was calculated as the difference between the event date (EVENT_DT) and the drug initiation date (START_DT). Records lacking complete date information (e.g., missing day) or with logical errors (e.g., START_DT later than EVENT_DT) were excluded to ensure data accuracy.

### 2.4 Logistic regression analysis

For multivariable logistic regression, candidate drugs were selected from the drugs identified in the disproportionality signal-detection analysis using a predefined sequential filtering strategy. Combination drug records were first excluded to avoid ambiguous attribution of risk to multiple active ingredients. Individual drugs were then retained if they met all predefined pharmacovigilance signal-strength criteria: ROR > 1, lower limit of the 95% CI > 1, and Bonferroni-adjusted P value < 0.01. The retained candidate drugs were entered into the multivariable logistic regression model together with demographic variables, including age, sex, and weight, to identify factors independently associated with OP-related adverse event reporting.

Age and weight were included as continuous variables, and sex was included as a categorical variable. Missing sex information was retained as an “unknown” category. Reports with missing age or weight values were excluded from the multivariable logistic regression analysis. A stepwise selection strategy based on the Akaike Information Criterion (AIC) was used to determine the final model. Model performance was evaluated using the area under the receiver operating characteristic curve (AUC).

### 2.5 Target prediction for PPIs

To explore potential mechanisms, we selected esomeprazole as the representative PPI for further analysis. Its standardized chemical structure was obtained from the PubChem database, and the Simplified Molecular Input Line Entry System (SMILES) notation was extracted for downstream target prediction. TargetNet and SwissTargetPrediction databases were queried, restricting the target organism to Homo sapiens, to identify potential drug–target interactions.

To improve prediction reliability, entries with incomplete structural data or missing results were removed, and duplicate targets were eliminated through cross-referencing. Target identifiers were further validated and annotated using the ChEMBL database, and gene names were standardized using UniProt to ensure nomenclature consistency.

A high-confidence target list was finalized for esomeprazole, providing a robust foundation for subsequent network toxicology and molecular docking analyses.

### 2.6 Identification of differentially expressed genes in OP

Two publicly available human transcriptome datasets, GSE56815 and GSE2208, were retrieved from the Gene Expression Omnibus (GEO) database (https://www.ncbi.nlm.nih.gov/geo/) based on the GPL96 platform. GSE56815 includes 40 high and 40 low BMD samples, while GSE2208 contains 10 high and 9 low BMD samples.

Raw expression data were normalized and log-transformed using the “limma” package in Bioconductor. To remove batch effects between datasets, we applied the “ComBat” function from the SVA package. Boxplots and principal component analysis (PCA) were generated before and after batch correction to assess sample consistency.

After integration and quality control, 99 valid samples were retained, including 50 normal controls and 49 OP cases. Differential expression analysis was conducted with thresholds of |log_2_ fold change| > 0.1 and p-value < 0.05. Genes meeting these criteria were considered differentially expressed genes (DEGs). A Venn diagram was used to identify overlapping genes between the DEGs and the predicted targets of esomeprazole, which were considered potential OP-specific targets for subsequent network analysis.

### 2.7 Functional enrichment analysis of target proteins

To investigate the potential biological functions of esomeprazole-associated targets in OP, Gene Ontology (GO) and Kyoto Encyclopedia of Genes and Genomes (KEGG) enrichment analyses were conducted. GO analysis was categorized into biological processes (BP), cellular components (CC), and molecular functions (MF), while KEGG pathway analysis focused on signaling pathways involved in bone metabolism, lipid regulation, and cytoskeletal remodeling.

A significance threshold of p < 0.05 was applied for all enrichment analyses. The results provided insights into the biological functions and pathways that may mediate the osteotoxicity of esomeprazole.

### 2.8 Protein-protein interaction network construction and hub gene identification

A protein-protein interaction network of the candidate genes overlapping between OP-related DEGs and esomeprazole targets was constructed using the STRING database, with the organism limited to Homo sapiens and the confidence score threshold set to >0.4. A statistical significance level of p < 1.0 × 10^-16^ was applied to ensure network robustness.

The resulting network was visualized using Cytoscape v3.10.1, and core regulatory targets (hub genes) were identified using the Degree algorithm in the CytoHubba plugin, based on node connectivity.

### 2.9 Molecular docking of core targets

To explore the potential binding interactions between esomeprazole and the hub proteins, molecular docking was performed using AutoDock Vina. The three-dimensional crystal structures of the core proteins were downloaded from the RCSB PDB database and preprocessed using PyMOL to remove water molecules and co-crystallized ligands. Protein structures were further prepared using AutoDock Tools 1.5.7 by adding hydrogen atoms, assigning Gasteiger charges, and merging non-polar hydrogens.

The chemical structure of esomeprazole was obtained from the PubChem database and converted to PDBQT format for docking. For each core target, the docking grid was defined to cover the predicted ligand-binding pocket. The detailed docking parameters, including receptor and ligand files, grid box center coordinates, box dimensions, exhaustiveness values, energy range, and number of binding modes generated, are provided in S3 Table. Binding poses and interacting residues were visualized using PyMOL to assess the computational binding plausibility between esomeprazole and the hub proteins.

### 2.10 Molecular dynamics simulation of core targets

Molecular dynamics (MD) simulations were conducted using GROMACS 2022 (https://manual.gromacs.org/2022/) to assess the conformational stability of protein–ligand complexes. The AMBER14SB force field was applied to proteins, and the General AMBER Force Field (GAFF) was used for ligands. The Transferable Intermolecular Potential with 3 Points (TIP3P) water model was employed, and systems were solvated in a periodic cubic box with added counterions for neutralization.

All hydrogen bonds were constrained using the Linear Constraint Solver (LINCS) algorithm, enabling a 2 fs time step. Long-range electrostatics were handled via the Particle Mesh Ewald (PME) method with a 1.2 nm cutoff. The van der Waals interaction cutoff was set to 10 Å and updated every 10 steps. Temperature was maintained at 298 K using the V-rescale thermostat, and pressure at 1 bar using the Berendsen barostat.

Following energy minimization, 100 ps equilibration was performed under constant number of particles, volume, and temperature (NVT) and constant number of particles, pressure, and temperature (NPT) conditions, respectively. Production MD simulations were then run for 100 ns, and snapshots were saved every 10 ps. The simulation trajectories were analyzed using Visual Molecular Dynamics (VMD) and the PyMOL Molecular Graphics System to evaluate structural stability and binding interactions over time.

### 2.11 Statistical analysis

All statistical analyses were performed using R software (v4.3.2), Microsoft Excel 2019, and MedDRA v26.1. Categorical variables were presented as counts and percentages, while continuous variables were expressed as medians with interquartile ranges (IQRs). Visualization and network analysis were conducted in Cytoscape v3.10.1, and data preprocessing scripts were executed in Perl v5.32.1. For FAERS disproportionality signal detection, statistical significance was assessed using Fisher’s exact test. The resulting p-values were adjusted using the Bonferroni correction to account for multiple drug-level comparisons, and the adjusted p-values (P-adjust) were used to assess the statistical significance of pharmacovigilance safety signals.

## 3. Results

### 3.1 Identification of OP-associated drugs

**Fig 1:**
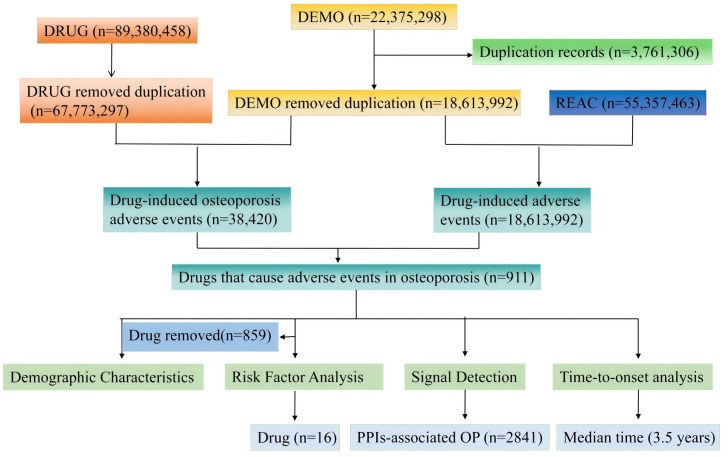
Flowchart of OP-related adverse event report selection.

From Q1 2004 to Q4 2024, a total of 55,357,463 AE reports were recorded in the FAERS database. After filtering, 38,420 reports were identified as being related to OP. The complete FAERS data extraction and filtering process is shown in Fig 1. Using four signal detection algorithms—ROR, PRR, BCPNN, and EBGM—we systematically analyzed drug–OP associations and identified 52 drugs with statistically significant signals.

Among these, the top 10 drugs with the highest number of OP-related reports were:

Tenofovir disoproxil (ROR = 156.74), Emtricitabine; Tenofovir disoproxil (ROR = 84.81), Esomeprazole (ROR = 19.05), Efavirenz; Emtricitabine; Tenofovir disoproxil (ROR = 98.07), Rituximab (ROR = 2.23), Medroxyprogesterone (ROR = 8.52), Prednisone (ROR = 5.48), Prednisolone (ROR = 5.00), Tocilizumab (ROR = 2.76), Emtricitabine; Rilpivirine; Tenofovir disoproxil (ROR = 79.11) (Table 1).

**Table 1:** Top 10 drugs associated with OP: A FAERS-based pharmacovigilance signal detection study.

Drug	N	ROR (95%Cl)	PRR (Chi-square)	EBGM(EBGM05)	IC (IC025)	p-adjust
Tenofovir Disoproxil	4707	156.74 (151.48 - 162.18)	122.22 (497625.05)	107.37 (104.35)	6.75 (6.7)	<0.001
Emtricitabine; Tenofovir Disoproxil	3072	84.81 (81.53 - 88.23)	73.15 (201564.65)	67.39 (65.19)	6.07 (6.02)	<0.001
Esomeprazole	2488	19.05 (18.28 - 19.85)	18.4 (38219.68)	17.28 (16.69)	4.11 (4.05)	<0.001
Efavirenz; Emtricitabine; Tenofovir Disoproxil	1589	98.07 (92.85 - 103.59)	82.26 (122534.93)	78.9 (75.37)	6.3 (6.22)	<0.001
Rituximab	473	2.23 (2.04 - 2.44)	2.22 (315.65)	2.21 (2.05)	1.14 (1.01)	<0.001
Medroxyprogesterone	404	8.52 (7.72 - 9.41)	8.39 (2608.91)	8.32 (7.66)	3.06 (2.91)	<0.001
Prednisone	392	5.48 (4.96 - 6.06)	5.43 (1405.34)	5.39 (4.95)	2.43 (2.28)	<0.001
Prednisolone	340	5 (4.49 - 5.56)	4.96 (1066.39)	4.92 (4.5)	2.3 (2.14)	<0.001
Tocilizumab	322	2.76 (2.48 - 3.09)	2.75 (357.58)	2.74 (2.5)	1.45 (1.29)	<0.001
Emtricitabine; Rilpivirine; Tenofovir Disoproxil	320	79.11 (70.26 - 89.06)	68.2 (21056.27)	67.64 (61.25)	6.08 (5.91)	<0.001

Notably, among all proton pump inhibitors (PPIs), esomeprazole ranked third overall, with 2,488 OP-related reports.

### 3.2 Risk factors for drug-induced OP

Among the 52 drugs with statistically significant OP-related signals, 9 combination drug records were excluded to avoid ambiguous attribution to multiple active ingredients. According to the predefined pharmacovigilance signal-strength criteria described in the Methods section, 16 candidate agents were retained for multivariable logistic regression analysis.

**Fig 2:**
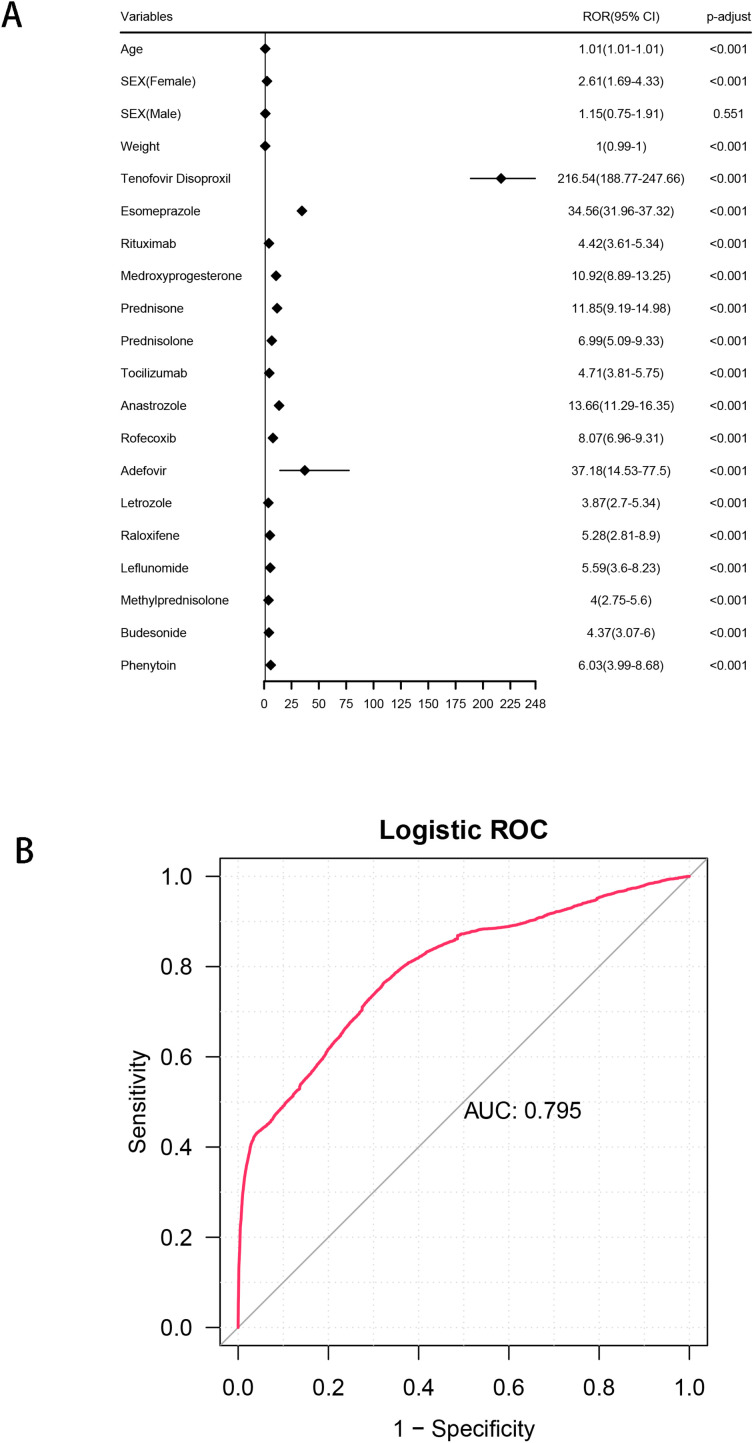
Identification and performance evaluation of independent risk factors for drug-induced OP. (A) Multivariable logistic regression analysis showing the adjusted ORs and 95% CIs for each risk factor. Bonferroni-corrected p-values (P-adjust) were used to determine statistical significance, with P-adjust < 0.01 considered significant. (B) ROC curve of the logistic regression model. An AUC greater than 0.7 indicates good model performance.

The multivariable logistic regression model incorporated these 16 candidate drug exposures and available demographic variables, including sex, age, and weight. After stepwise model optimization based on the AIC, the final model identified female sex, advanced age, low body weight, and exposure to tenofovir disoproxil, esomeprazole, rituximab, medroxyprogesterone, and prednisone as factors independently associated with OP-related adverse event reporting (Fig 2A). The model showed good discriminative performance, with an area under the receiver operating characteristic curve of 0.795 (Fig 2B).

### 3.3 Baseline characteristics and signal detection of PPIs

A total of 2,841 PPI-associated OP-related reports were identified in the FAERS database. Among these reports, esomeprazole was the most frequently reported PPI, accounting for 87.6% (N = 2,488) of all PPI-associated OP-related reports. This proportion should be interpreted as a reporting distribution within FAERS rather than an exposure-adjusted incidence or direct estimate of intrinsic OP risk. The remaining reports involved omeprazole (N = 158), pantoprazole (N = 108), lansoprazole (N = 43), rabeprazole (N = 17), and dexlansoprazole (N = 16) (Table 2).

**Table 2:** Demographic and outcome characteristics of patients using PPIs.

Variables	Esomeprazole	Omeprazole	Pantoprazole	Lansoprazole	Rabeprazole	Dexlansoprazole
N	2488	158	108	43	17	16
Gender						
Female	1447 (58.2%)	98 (62.0%)	77 (71.3%)	34 (79.1%)	13 (76.5%)	8 (50.0%)
Male	423 (17.0%)	46 (29.1%)	24 (22.2%)	6 (14.0%)	4 (23.5%)	6 (37.5%)
Unknown	618 (24.8%)	14 (8.9%)	7 (6.5%)	3 (7.0%)	0 (0%)	2 (12.5%)
Weight						
<50 kg	50 (2.0%)	9 (5.7%)	3 (2.8%)	1 (2.3%)	1 (5.9%)	1 (6.3%)
>100 kg	159 (6.4%)	5 (3.2%)	4 (3.7%)	0 (0%)	0 (0%)	0 (0%)
50–100 kg	753 (30.3%)	73 (46.2%)	45 (41.7%)	18 (41.9%)	4 (23.5%)	6 (37.5%)
Unknown	1526 (61.3%)	71 (44.9%)	56 (51.9%)	24 (55.8%)	12 (70.6%)	9 (56.3%)
Age						
<18 years	3 (0.1%)	0 (0%)	0 (0%)	2 (4.7%)	0 (0%)	0 (0%)
>85 years	21 (0.8%)	13 (8.2%)	3 (2.8%)	6 (14.0%)	1 (5.9%)	0 (0%)
18–65 years	1182 (47.5%)	49 (31.0%)	50 (46.3%)	16 (37.2%)	4 (23.5%)	2 (12.5%)
65–85 years	334 (13.4%)	56 (35.4%)	25 (23.1%)	15 (34.9%)	8 (47.1%)	4 (25.0%)
Unknown	948 (38.1%)	40 (25.3%)	30 (27.8%)	4 (9.3%)	4 (23.5%)	10 (62.5%)
Reporter Country						
United States	2434 (97.8%)	72 (45.6%)	34 (31.5%)	9 (20.9%)	10 (58.8%)	16 (100%)
Canada	16 (0.2%)		24 (22.2%)		3 (17.6%)	
France	12 (0.1%)				2 (11.8%)	
United Kingdom		38 (24.1%)		18 (41.9%)		
Poland		9 (5.7%)				
Germany			24 (22.2%)			
Japan				4 (9.3%)		
Outcome						
Death	42 (1.7%)	0 (0%)	6 (5.6%)	1 (2.3%)	0 (0%)	0 (0%)
Disability	238 (9.6%)	20 (12.7%)	7 (6.5%)	2 (4.7%)	0 (0%)	1 (6.3%)
Hospitalization	758 (30.5%)	36 (22.8%)	46 (42.6%)	23 (53.5%)	5 (29.4%)	1 (6.3%)
Life-Threatening	5 (0.2%)	4 (2.5%)	1 (0.9%)	0 (0%)	0 (0%)	0 (0%)
Other	1445 (58.1%)	98 (62.0%)	48 (44.4%)	17 (39.5%)	12 (70.6%)	14 (87.5%)
Serious Cases						
No	1193 (48.0%)	50 (31.6%)	22 (20.4%)	6 (14.0%)	11 (64.7%)	7 (43.8%)
Yes	1295 (52.0%)	108 (68.4%)	86 (79.6%)	37 (86.0%)	6 (35.3%)	9 (56.3%)
Fatal Cases						
No	2446 (98.3%)	158 (100%)	102 (94.4%)	42 (97.7%)	17 (100%)	16 (100%)
Yes	42 (1.7%)	0 (0%)	6 (5.6%)	1 (2.3%)	0 (0%)	0 (0%)
Reporter Type						
Consumer	500 (20.1%)	85 (53.8%)	50 (46.3%)	15 (34.9%)	8 (47.1%)	7 (43.8%)
Health Professional	5 (0.2%)	12 (7.6%)	15 (13.9%)	4 (9.3%)	4 (23.5%)	0 (0%)
Pharmacist	8 (0.3%)	7 (4.4%)	3 (2.8%)	5 (11.6%)	1 (5.9%)	0 (0%)
Physician	27 (1.1%)	26 (16.5%)	21 (19.4%)	6 (14.0%)	1 (5.9%)	5 (31.3%)
Missing	1948 (78.3%)	28 (17.7%)	19 (17.6%)	13 (30.2%)	3 (17.6%)	4 (25.0%)

**Fig 3:**
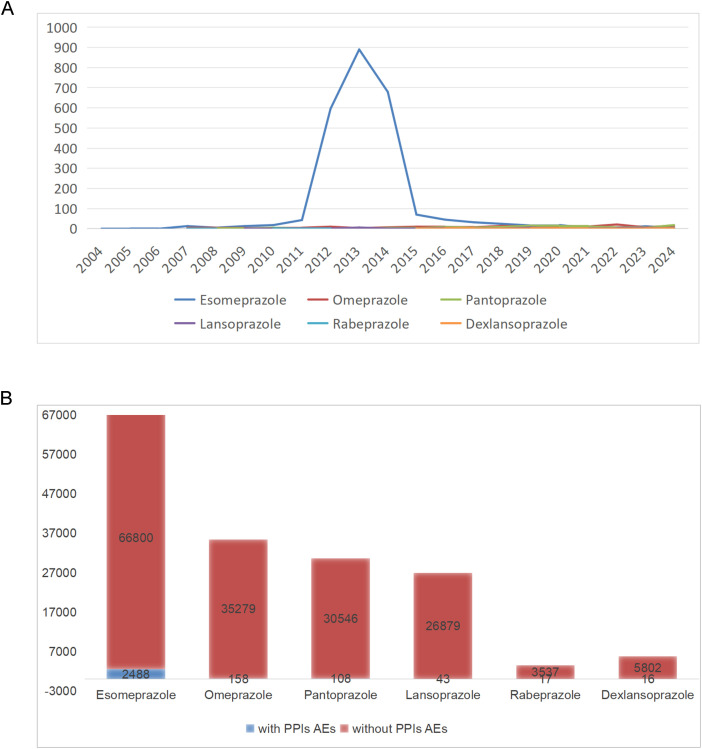
Time trends and case distribution of esomeprazole-associated OP reports in FAERS. (A) Quarterly reporting rate of OP events associated with esomeprazole from Q1 2004 to Q4 2024 in the FAERS database. (B) Comparison of the number of reports involving esomeprazole with versus without co-reported OP.

As shown in Fig 3A, esomeprazole-related OP reports increased rapidly beginning in 2012, peaking between 2013 and 2014, and subsequently declining over time. Other PPIs, such as omeprazole and pantoprazole, showed mild increases in recent years but remained far below esomeprazole in total report volume. Fig 3B shows that the reporting proportion of OP-related AEs among esomeprazole-associated reports was 3.59% (2,488/69,288), which was higher than the corresponding reporting proportions for omeprazole (0.45%) and pantoprazole (0.35%). However, because FAERS lacks reliable exposure denominators, these values should not be interpreted as true incidence rates or exposure-adjusted risks.

In terms of demographic characteristics(Table 2), females were disproportionately represented across all PPI-associated reports. Notably, lansoprazole and rabeprazole cases had female proportions of 79.1% and 76.5%, respectively. The predominant age group was 18–65 years, with esomeprazole accounting for 47.5% of cases in this range. Although weight data were largely missing, available records suggested that most patients weighed between 50–100 kg.

Geographically, esomeprazole-related reports were primarily from the United States (97.8%), while reports for other PPIs displayed greater international variability. Consumers were the main reporters for most drugs, although many entries lacked source information.

Regarding outcomes, 52.0% of esomeprazole reports were classified as serious AEs, with a mortality rate of 1.7%. Although pantoprazole had a higher mortality rate (5.6%), the small sample size warrants cautious interpretation. Hospitalization rates were also high for pantoprazole (42.6%) and lansoprazole (53.5%).

We further assessed the signal strength of each PPI using all four detection algorithms (Table 3). Only esomeprazole showed positive signals across all methods, with a chi-square value of 28,817.66 and a Bonferroni-adjusted p-value of 0, indicating a strong and statistically robust association with OP. In contrast, omeprazole, pantoprazole, and rabeprazole yielded positive signals under selected methods, while lansoprazole and dexlansoprazole did not show significant signals.

**Table 3:** Disproportionality signal detection results for osteoporosis associated with PPIs in FAERS.

PPI Name	N	Positive Signal				Х^2^	Bonferroni-corrected P-value
		(ROR)	(PRR)	(MGPS)	(BCPNN)		
Esomeprazole	2488	Yes	Yes	Yes	Yes	28817.66274	0
Omeprazole	158	Yes	No	No	Yes	49.49403601	3.97951 × 10^−12^
Pantoprazole	108	Yes	No	No	Yes	16.41896832	0.000101549
Lansoprazole	43	No	No	No	No	6.475420376	0.021875288
Rabeprazole	17	Yes	Yes	No	Yes	14.28776023	0.000313763
Dexlansoprazole	16	No	No	No	No	1.315217851	0.502904207
Total	2841	Yes	Yes	Yes	Yes	13579.55977	0

In summary, based on the number of reports, temporal trends, reporting proportions, and multidimensional signal strength analysis, esomeprazole exhibited the strongest association with OP-related adverse event reporting among PPIs. Therefore, subsequent network toxicology and molecular docking analyses focused on esomeprazole to further elucidate its potential molecular mechanisms and key regulatory targets.

### 3.4 TTO between drug exposure and OP

**Fig 4:**
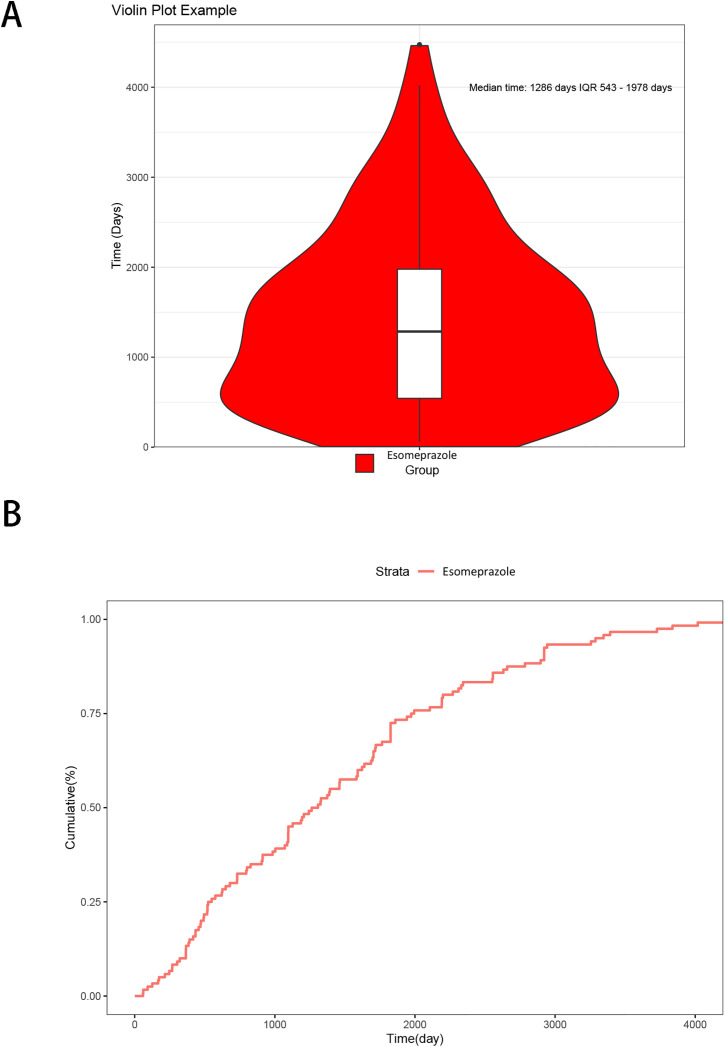
Onset time analysis of osteoporosis-related AEs. (A) Violin plot illustrating the overall TTO distribution of drug-induced OP. (B) Cumulative incidence curve showing the temporal distribution of OP-related adverse events across all identified drugs.

To further explore the temporal relationship between PPI administration and the onset of OP, we calculated the time interval between the start of drug exposure (START_DT) and the occurrence of adverse events (EVENT_DT). The median latency time was 3.5 years (interquartile range [IQR]: 1.5–5.4 years) (Fig 4).

In addition, Weibull distribution modeling revealed a shape parameter (β) greater than 1, indicating a wear-out failure pattern with an increasing hazard over time. This suggests that OP-related adverse events associated with PPIs may be more likely to occur after sustained or prolonged exposure rather than primarily at the early stage of treatment (Table 4).

**Table 4:** TTO analysis of esomeprazole-associated osteoporosis based on weibull distribution.

scale parameter		shape parameter		Type
α	95%CI	β	95%CI	Wear-out failure
1579.9	(1373.8-1786.0)	1.44	(1.24-1.65)	

### 3.5 Chemical characteristics and target identification of esomeprazole

The chemical information of esomeprazole, including molecular formula, molecular weight, and SMILES structure, was retrieved from the PubChem database (S4 Table) to support subsequent computational modeling.

Using SwissTargetPrediction and TargetNet, and after removing redundant or incomplete entries, we identified 475 unique potential protein targets. This provided a comprehensive target profile database for esomeprazole, enabling further mechanistic exploration.

### 3.6 Identification of DEGs in OP

Two human bone density-related microarray datasets based on the GPL96 platform—GSE56815 and GSE2208—were downloaded and integrated from the GEO database, comprising a total of 99 samples (49 OP and 50 healthy controls).

**Fig 5:**
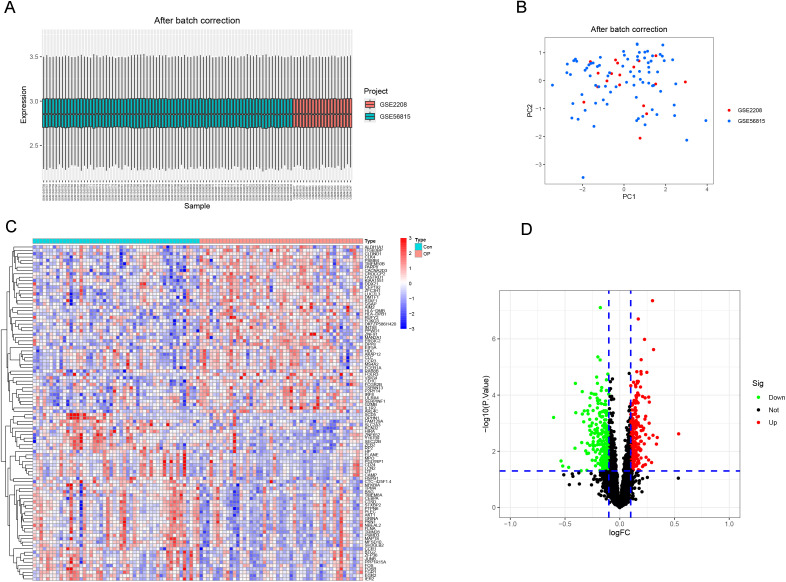
Batch effect correction and differential expression analysis of OP-related genes. (A) Box plot showing the expression distribution of the merged dataset after batch effect correction. (B) PCA plot of the corrected dataset, colored by sample group. (C) Heatmap of DEGs between OP samples and healthy controls. (D) Volcano plot visualizing the DEGs between OP and control groups, highlighting significantly up- and downregulated genes.

Data preprocessing involved normalization and log-transformation using the limma package, followed by batch effect correction with the ComBat function in the SVA package. Boxplots (Fig 5A) and PCA plots (Fig 5B) after batch correction confirmed adequate data harmonization.

Differential expression analysis identified 523 DEGs, including 240 upregulated and 283 downregulated genes (Fig 5C and 5D), based on the threshold of log_2_FC > 0.1 and p < 0.05.

### 3.7 Functional enrichment and pathway analysis of overlapping genes

**Fig 6:**
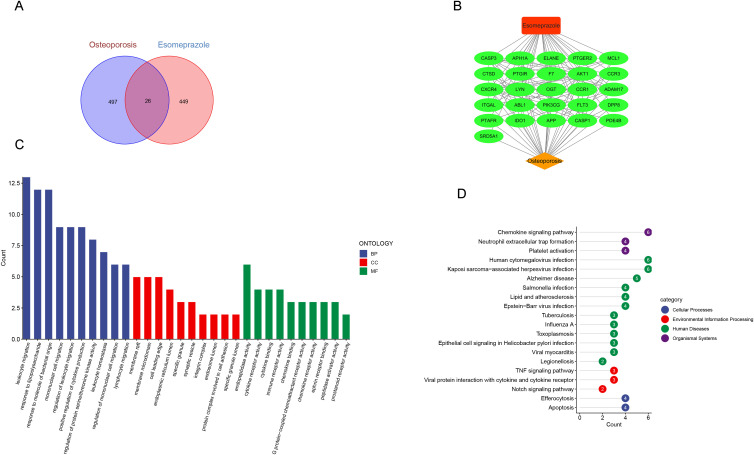
Identification of common targets and enrichment analysis between esomeprazole and osteoporosis. (A) Venn diagram showing the overlapping genes between predicted targets of esomeprazole and DEGs associated with OP. (B) Network visualization of shared targets between esomeprazole and OP. Green circles represent genes, the orange quadrilateral represents OP, and red-bordered nodes indicate esomeprazole. (C) GO enrichment analysis of the 26 shared genes. The bar chart displays the number of genes enriched in each term across three categories: BP, CC, and MF. (D) KEGG pathway enrichment analysis of shared genes. The lollipop chart indicates the number of enriched genes in each pathway, with different colors representing distinct KEGG categories.

By intersecting the 475 predicted esomeprazole targets with the 523 OP-related DEGs, we identified 26 overlapping genes, considered as putative effectors linking esomeprazole exposure to OP development (Fig 6A).

An interaction map illustrating the potential regulatory cascade from esomeprazole exposure → gene modulation → OP was constructed (Fig 6B), providing exploratory clues regarding potential drug-related biological processes.

GO enrichment analysis revealed that these genes were mainly involved in: BP: leukocyte migration, responses to lipopolysaccharide and bacterial molecules; CC: membrane microdomains, cell leading edge, endoplasmic reticulum lumen; MF: chemokine binding and G-protein-coupled chemoattractant receptor activity (Fig 6C).

KEGG pathway analysis indicated significant enrichment in several bone-related signaling cascades, including Notch, cAMP, neurotrophin, AGE-RAGE, and MAPK pathways (Fig 6D), suggesting a possible network associated with esomeprazole-related bone metabolic processes.

### 3.8 Protein-protein interaction network construction and hub gene identification

**Fig 7:**
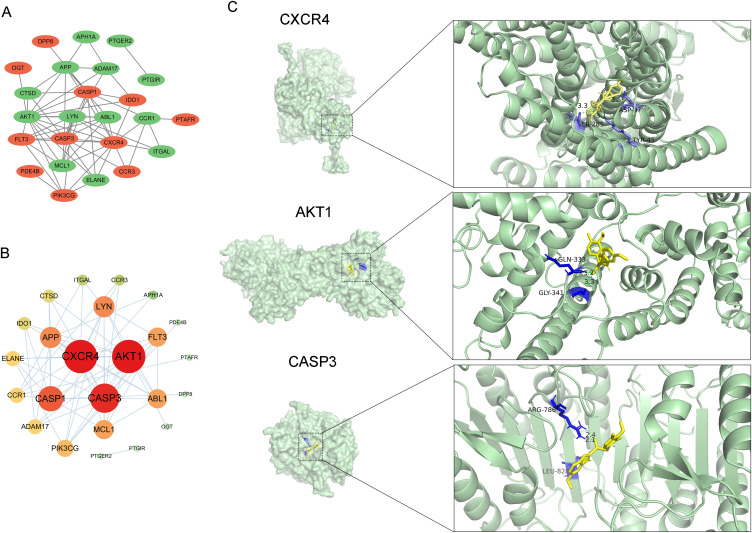
Protein-protein interaction network construction, hub gene identification, and molecular docking of esomeprazole with core targets. (A) Protein–protein interaction network of key genes associated with esomeprazole-related OP biological processes. red nodes represent upregulated genes; green nodes represent downregulated genes. (B) Core protein-protein interaction network construction derived from the degree centrality algorithm, highlighting hub genes shared between esomeprazole targets and OP-related DEGs. (C) Molecular docking models showing predicted binding poses of esomeprazole with the three candidate hub proteins: CXCR4, AKT1, and CASP3. Binding poses and interaction sites are displayed in 3D visualization.

To examine protein–protein interactions among the 26 key genes, we constructed a protein-protein interaction network using the STRING database (confidence score > 0.4, p < 1.0 × 10^-16^), restricted to Homo sapiens. The network was visualized using Cytoscape, with red and green nodes representing up- and downregulated genes, respectively (Fig 7A).

Core regulatory nodes were identified using the degree centrality algorithm from the CytoHubba plugin. The top three hub genes were CXCR4, AKT1, and CASP3 (Fig 7B), which were highly interconnected and may represent candidate hub genes linking esomeprazole exposure to OP-related biological processes.

### 3.9 Molecular docking analysis of esomeprazole and core targets

Molecular docking was performed to evaluate the computational binding plausibility between esomeprazole and the candidate hub proteins. Using the docking parameters summarized in S3 Table, esomeprazole showed favorable predicted docking scores with CXCR4, AKT1, and CASP3, with predicted binding energies of −8.1, −8.1, and −7.9 kcal/mol, respectively. In molecular docking studies, predicted binding energies around −7 to −9 kcal/mol are generally considered favorable, suggesting plausible ligand–target interactions. Visual inspection of the docking poses (Fig 7C) revealed potential hydrogen-bonding interactions between esomeprazole and key residues of the hub proteins. Specifically, esomeprazole formed predicted hydrogen bonds with SER-285, ASP-97, and TYR-45 in CXCR4; GLN-333 and GLY-341 in AKT1; and ARG-786 and LEU-828 in CASP3.

These predicted interactions provide computational support for binding plausibility, but they do not demonstrate functional modulation of these targets. Therefore, these targets should be interpreted as hypothesis-generating candidate targets requiring further experimental validation.

### 3.10 Molecular dynamics simulation of core protein–ligand complexes

To further assess the dynamic stability between esomeprazole and its core targets, we performed 100 ns MD simulations for three protein–ligand complexes: CXCR4, AKT1, and CASP3. Key structural parameters—including root mean square deviation (RMSD), radius of gyration (Rg), center-of-mass (COM) distance, and buried solvent-accessible surface area (buried SASA)—were analyzed to evaluate interaction strength and conformational stability.

**Fig 8:**
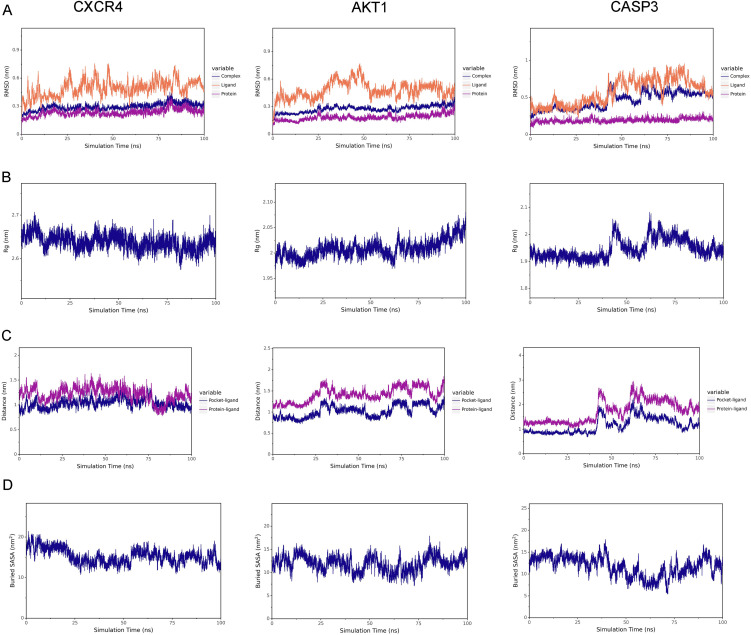
Structural stability and binding behavior of esomeprazole–target complexes during 100 ns molecular dynamics simulations. (A) RMSD plots showing the overall conformational stability of the protein–ligand complexes over the simulation time. (B) Rg plots indicating the compactness and structural integrity of the protein–ligand complexes. (C)COM distance between the ligand and its respective protein or binding site, reflecting the stability of the binding interactions. (D) Buried SASA quantifying the degree of ligand encapsulation within the protein binding pocket.

As shown in Fig 8, the RMSD values (top row) for all complexes remained within 0.2–0.5 nm throughout the simulation period, suggesting that the overall conformations were stable. Among the three, the CXCR4–esomeprazole complex exhibited the lowest RMSD fluctuation, indicating minimal deviation from its initial docked pose and the highest conformational stability.

Rg values (second row) stabilized after approximately 20 ns for all complexes, indicating that no major unfolding events occurred during ligand binding. Notably, the AKT1 complex displayed slightly lower Rg values, suggesting a more compact structure.

The center-of-mass distances between esomeprazole and both the whole protein and binding pocket (third row) rapidly decreased during the early phase of simulation and plateaued thereafter. This suggested that the ligand remained close to its initial binding site and did not dissociate. The CASP3 complex showed the most consistent pocket–ligand distance, highlighting a particularly stable binding interface.

Buried SASA values (bottom row) exceeded 10 nm² across all three complexes, reflecting robust contact surface areas between ligand and protein. The CXCR4 complex showed the highest buried SASA (~20 nm²), indicating a deeply embedded interaction interface and a relatively stable protein-ligand contact interface.

Collectively, the MD simulations suggested that the predicted esomeprazole-target complexes maintained relatively stable conformations during the 100-ns simulations. These results support conformational stability but should not be interpreted as quantitative validation of binding affinity or target modulation.

## 4. Discussion

Based on large-scale real-world data extracted from the FDA Adverse Event Reporting System, this study identified a total of 38,420 OP-related adverse event reports. Among all implicated drugs, esomeprazole ranked third in terms of reporting frequency. After predefined pharmacovigilance signal-based filtering, 16 candidate drugs were included in the multivariable logistic regression model. The final model showed that advanced age, female sex, low body weight, and exposure to several drugs, including tenofovir disoproxil, esomeprazole, rituximab, medroxyprogesterone, and prednisone, were independently associated with OP-related adverse event reporting.

These findings are consistent with prior studies. Postmenopausal women, in particular, exhibit accelerated bone turnover and increased risk of bone loss due to estrogen deficiency, rendering them more susceptible to bone metabolic disorders [17,18]. In addition, low body weight has been recognized as a significant risk factor for OP, likely due to reduced skeletal loading and increased sensitivity to bone metabolic disruption [19,20]. Our study also showed that OP-related adverse event reports were more frequently observed in middle-aged and older populations, aligning with known epidemiological patterns of age-related bone loss [21,22]. These results support the need for careful risk assessment when prescribing medications with potential skeletal effects, particularly in older adults and other high-risk populations.

Among the candidate drugs included in the multivariable regression analysis, several known bone-affecting agents, including glucocorticoids and hormone-related drugs, showed associations with OP-related adverse event reporting, which is consistent with their recognized skeletal effects [23]. Notably, esomeprazole was independently associated with OP-related reporting and showed the strongest PPI-related pharmacovigilance signal, accounting for 87.6% of PPI-associated OP-related reports. It also exhibited consistent positive signals across four major signal detection algorithms—ROR, PRR, BCPNN, and EBGM—supporting its prioritization for further network toxicology analysis. However, this high reporting proportion should be interpreted cautiously, as it may be influenced by prescription volume, market share, drug availability, utilization patterns, and reporting bias rather than reflecting a higher intrinsic biological risk.

Mechanistically, PPIs are thought to contribute to OP primarily by chronically suppressing gastric acid secretion, thereby reducing calcium ion solubility and impairing intestinal calcium absorption [24]. This disturbance in calcium homeostasis and bone remodeling may ultimately lead to reduced bone mineral density and compromised bone microarchitecture [25]. Numerous studies have reported that long-term PPI use is associated with a 20%–50% increased risk of hip and vertebral fractures, with elderly individuals being particularly vulnerable [26]. In this study, time-to-onset analysis provided useful temporal information on OP-related adverse event reporting after PPI exposure. The median latency of 3.5 years suggests that OP-related events were often reported after relatively long-term PPI use, which is clinically relevant given the widespread chronic use of these agents. However, TTO in FAERS reflects the reported interval between drug initiation and adverse event onset, rather than a precise measure of cumulative exposure or dose-response effects. Because information on drug dose, treatment frequency, adherence, and cumulative exposure is incomplete in FAERS, the present study could not reliably evaluate dose-dependent or duration-dependent associations. Nevertheless, the temporal reporting pattern supports the need for greater attention to bone health monitoring in long-term PPI users, especially in populations with pre-existing osteoporosis risk factors, and highlights the value of future population-based studies with detailed exposure data.

To further explore the potential molecular links between esomeprazole and OP-related biological processes, we integrated predicted esomeprazole targets with OP-associated DEGs. This analysis yielded 26 overlapping genes, which were considered exploratory candidate targets for subsequent network toxicology analysis. GO and KEGG enrichment analyses showed that these genes were enriched in biological processes and signaling pathways relevant to bone metabolism and inflammation, including leukocyte migration, lipopolysaccharide response, chemokine signaling, Notch signaling, MAPK signaling, and AGE-RAGE signaling pathways. Previous studies have shown that disturbances in stem cell migration, inflammatory responses, and signaling cascades can impair bone regeneration and homeostasis, ultimately contributing to reduced bone mass and mineral density [27]. These findings suggest that the identified candidate genes may be associated with biological processes relevant to bone metabolic regulation; however, whether esomeprazole directly affects these processes requires further experimental validation.

Further construction of a protein-protein interaction network identified CXCR4, AKT1, and CASP3 as top hub genes, suggesting that they may represent key computational nodes linking esomeprazole exposure to OP-related biological processes. CXCR4 is involved in bone marrow-derived mesenchymal stem cell migration and osteoclast activity, and previous studies have linked CXCR4-related signaling to osteogenic differentiation, bone remodeling, and osteoporosis-related phenotypes [28–30]. AKT1, a central component of the PI3K/AKT pathway, is important for osteoblast proliferation, survival, and differentiation, and activation of AKT-related signaling has been reported to promote osteogenesis and alleviate bone loss [31,32]. CASP3 is a key effector of apoptosis, and increased CASP3 activity may promote osteoblast apoptosis and impair bone formation [33,34]. These findings support the biological relevance of the identified hub genes in bone metabolism.

Molecular docking and MD simulations provided additional computational support for the predicted interactions between esomeprazole and CXCR4, AKT1, and CASP3. The three complexes showed favorable predicted docking energies below −7.9 kcal/mol and maintained relatively stable conformations during the 100-ns simulations. Among them, the CXCR4-esomeprazole complex showed comparatively favorable dynamic stability, as reflected by stable RMSD profiles, compact Rg values, and relatively large buried SASA values. However, docking and MD simulations cannot determine whether esomeprazole functionally modulates these proteins or affects bone metabolism in biological systems. Therefore, these results should be interpreted as hypothesis-generating computational evidence rather than definitive proof of target modulation or biological toxicity.

Collectively, our results suggest that esomeprazole-associated OP-related reporting may involve a multi-target and multi-pathway network related to CXCR4, AKT1, and CASP3, as well as MAPK, Notch, cAMP, and PI3K/AKT-related signaling. From a network toxicology perspective, bone metabolic homeostasis is regulated by coordinated interactions among osteoblasts, osteoclasts, osteocytes, bone marrow-derived mesenchymal stem cells, immune-inflammatory mediators, apoptotic regulators, and endocrine-metabolic signals. Therefore, drug-associated bone toxicity is unlikely to result from a single molecular event, but may arise from disturbances across interconnected biological networks [35].

Classical drug-induced OP models support the concept of multi-network skeletal toxicity. Glucocorticoids mainly impair osteoblast differentiation and bone resorption-related signaling [36], whereas antiretroviral agents such as tenofovir may affect mineral metabolism and bone remodeling [37]. In contrast, PPIs have traditionally been linked to impaired calcium absorption due to gastric acid suppression. Our findings provide additional network toxicology-based clues suggesting that PPI-associated bone toxicity may also involve inflammatory, chemokine-related, apoptotic, and osteogenic signaling processes. By integrating pharmacovigilance signals, predicted drug targets, disease-related transcriptomic changes, protein-protein interaction networks, enrichment analysis, molecular docking, and MD simulations, network computational pharmacology can help prioritize candidate targets and generate mechanistic hypotheses. Recent OP studies using similar strategies further support their value in identifying candidate intervention targets and exploring drug-repurposing opportunities [38]. Nevertheless, these findings should be interpreted as computational hypotheses rather than experimentally validated mechanisms.

From a clinical perspective, these findings may help raise awareness of potential bone health concerns in long-term PPI users, particularly those with established OP risk factors such as advanced age, female sex, low body weight, prior fracture history, baseline low bone mass, or concomitant use of bone-affecting medications. The results do not support indiscriminate discontinuation of PPIs, but they may support individualized risk assessment, periodic reassessment of the need for long-term PPI therapy, and appropriate bone health monitoring according to patient-specific risk profiles. Further population-based studies with detailed exposure and clinical outcome data are needed to validate the observed pharmacovigilance signals.

Despite the comprehensive integration of real-world pharmacovigilance data, bioinformatics analysis, and computational toxicology to explore the potential mechanisms underlying esomeprazole-associated OP-related adverse event reporting, this study has several notable limitations.

First, FAERS is a spontaneous reporting system. Although it provides large-scale data and valuable signal detection potential, its data quality is affected by reporter subjectivity, voluntary reporting, missing information, and heterogeneous exposure backgrounds, which may introduce reporting bias and limit causal inference. In addition, FAERS provides limited patient-level clinical information. Although age, sex, and weight were included in the multivariable logistic regression model, other important OP-related factors, such as lifestyle factors, genetic predisposition, baseline bone health status, comorbidities, and treatment details, were not consistently available and could not be adjusted for. These unmeasured factors may have acted as residual confounders.

Second, the network toxicology analysis relied mainly on public databases, DEG screening, and in silico prediction models. The DEG screening strategy was relatively permissive, using |log2FC| > 0.1 and nominal p < 0.05 without FDR-adjusted p values as the primary filtering criterion. Although this discovery-oriented threshold was selected to capture modest transcriptomic changes in OP-related microarray datasets, it may introduce false-positive background signals. Sensitivity analyses using stricter thresholds retained only a limited number of DEGs, which were insufficient for stable downstream network analysis. In addition, predicted esomeprazole targets derived from public databases may also contain false-positive associations. Therefore, the overlapping genes should be interpreted as exploratory candidate targets rather than validated disease-driving genes.

Third, the proposed molecular mechanisms have not been experimentally validated. Molecular docking and MD simulations can support binding plausibility and conformational stability, but they cannot confirm whether esomeprazole functionally regulates CXCR4, AKT1, or CASP3. Moreover, the MD analysis was limited to geometric stability indicators, including RMSD, Rg, COM distance, and buried SASA, without MM-PBSA/MM-GBSA binding free energy calculations, residue-level energy decomposition, or replicate simulations. Therefore, the docking and MD results should be interpreted as exploratory computational evidence rather than quantitative thermodynamic validation or definitive proof of target modulation. Future studies using larger transcriptomic cohorts, bone-related cell and animal models, target-specific assays, binding free energy calculations, and replicated MD simulations are needed to validate these findings.

## 5. Conclusion

In this study, FAERS analysis identified 52 drugs with potential OP-related reporting signals, among which esomeprazole showed consistent signals across multiple detection algorithms. Integrated network toxicology and molecular modeling identified 26 candidate targets and highlighted MAPK, Notch, and cAMP pathways, together with CXCR4, AKT1, and CASP3, as computational clues linking esomeprazole to OP-related biological processes. Molecular docking and MD simulations supported the binding plausibility and conformational stability of the predicted esomeprazole–target complexes. These findings provide exploratory mechanistic clues and support further experimental validation of the identified targets and pathways.

## Supporting information

S1 Table 2 × 2 table for disproportionality analysis. (DOCX)

S2 Table Signal detection methods, formulas, and generation criteria. (DOCX)

S3 Table AutoDock Vina docking parameters for esomeprazole with core target proteins. (DOCX)

S4 Table Chemical information of esomeprazole. (DOCX)

## Abbreviations

3D: Three-dimensional
AE: Adverse Event
AIC: Akaike Information Criterion
AUC: Area Under the Receiver Operating Characteristic Curve
BCPNN: Bayesian Confidence Propagation Neural Network
BMD: Bone Mineral Density
BMSC: Bone Marrow-Derived Mesenchymal Stem Cell
BP: Biological Processes
CC: Cellular Components
COM: Center-of-Mass
DEG: Differentially Expressed Gene
EBGM: Empirical Bayes Geometric Mean
FAERS: FDA Adverse Event Reporting System
FDA: Food and Drug Administration
GAFF: General AMBER Force Field
GEO: Gene Expression Omnibus
GO: Gene Ontology
IQR: Interquartile Range
KEGG: Kyoto Encyclopedia of Genes and Genomes
LINCS: Linear Constraint Solver
MD: Molecular Dynamics
MF: Molecular Functions
NPT: Number of Particles, Pressure, and Temperature
NVT: Number of Particles, Volume, and Temperature
OP: Osteoporosis
PCA: Principal Component Analysis
PPIs: Proton Pump Inhibitors
PME: Particle Mesh Ewald
PRR: Proportional Reporting Ratio
PT: Preferred Term
Rg: Radius of Gyration
RMSD: Root Mean Square Deviation
ROR: Reporting Odds Ratio
SASA: Solvent-Accessible Surface Area
SMILES: Simplified Molecular Input Line Entry System
TIP3P: Transferable Intermolecular Potential with 3 Points
TTO: Time to Onset
VMD: Visual Molecular Dynamics
WHO: World Health Organization

## References

[1] Lane NE. Epidemiology, etiology, and diagnosis of osteoporosis. Am J Obstet Gynecol. 2006;194(2 Suppl):S3-11. doi: 10.1016/j.ajog.2005.08.047 16448873

[2] Gopinath V. Osteoporosis. Med Clin N Am. 2023;107(2):213–25. doi: 10.1016/j.mcna.2022.10.013 36759092

[3] Kanis JA. Diagnosis of osteoporosis and assessment of fracture risk. Lancet. 2002;359(9321):1929–36. doi: 10.1016/S0140-6736(02)08761-5 12057569

[4] Snega Priya P, Pratiksha Nandhini P, Arockiaraj J. A comprehensive review on environmental pollutants and osteoporosis: insights into molecular pathways. Environ Res. 2023;237(Pt 2):117103. doi: 10.1016/j.envres.2023.117103 37689340

[5] Hernlund E, Svedbom A, Ivergård M, Compston J, Cooper C, Stenmark J, et al. Osteoporosis in the European Union: medical management, epidemiology and economic burden. A report prepared in collaboration with the International Osteoporosis Foundation (IOF) and the European Federation of Pharmaceutical Industry Associations (EFPIA). Arch Osteoporos. 2013;8(1):136. doi: 10.1007/s11657-013-0136-1 24113837 PMC3880487

[6] Nguyen K-D, Bagheri B, Bagheri H. Drug-induced bone loss: a major safety concern in Europe. Expert Opin Drug Saf. 2018;17(10):1005–14. doi: 10.1080/14740338.2018.1524868 30222369

[7] Bioletto F, Pusterla A, Fraire F, Sauro L, Presti M, Arvat E, et al. Sex-specific association of chronic proton pump inhibitor use with reduced bone density and quality. J Clin Endocrinol Metab. 2025;110(6):e2071–9. doi: 10.1210/clinem/dgae598 39197024 PMC12086406

[8] Targownik LE, Lix LM, Metge CJ, Prior HJ, Leung S, Leslie WD. Use of proton pump inhibitors and risk of osteoporosis-related fractures. CMAJ. 2008;179(4):319–26. doi: 10.1503/cmaj.071330 18695179 PMC2492962

[9] Yang Y-X, Lewis JD, Epstein S, Metz DC. Long-term proton pump inhibitor therapy and risk of hip fracture. JAMA. 2006;296(24):2947–53. doi: 10.1001/jama.296.24.2947 17190895

[10] Wright MJ, Proctor DD, Insogna KL, Kerstetter JE. Proton pump-inhibiting drugs, calcium homeostasis, and bone health. Nutr Rev. 2008;66(2):103–8. doi: 10.1111/j.1753-4887.2008.00015.x 18254877

[11] Bioletto F, Pusterla A, Fraire F, Sauro L, Presti M, Arvat E, et al. Sex-specific association of chronic proton pump inhibitor use with reduced bone density and quality. J Clin Endocrinol Metab. 2025;110(6):e2071–9. doi: 10.1210/clinem/dgae598 39197024 PMC12086406

[12] Bahtiri E, Islami H, Hoxha R, Qorraj-Bytyqi H, Rexhepi S, Hoti K, et al. Esomeprazole use is independently associated with significant reduction of BMD: 1-year prospective comparative safety study of four proton pump inhibitors. J Bone Miner Metab. 2016;34(5):571–9. doi: 10.1007/s00774-015-0699-6 26209167

[13] Hansen KE, Nieves JW, Nudurupati S, Metz DC, Perez MC. Dexlansoprazole and esomeprazole do not affect bone homeostasis in healthy postmenopausal women. Gastroenterology. 2019;156(4):926-934.e6. doi: 10.1053/j.gastro.2018.11.023 30445008

[14] Belmont AP, Novotny S, Mukherjee EM, Park D, Wong KH, Martin-Pozo MD, et al. Anaphylaxis to Penicillin: harvesting insights from the food and drug administration’s adverse event reporting system. J Allergy Clin Immunol Pract. 2025;13(12):3243–53. doi: 10.1016/j.jaip.2025.07.039 40752738 PMC12926993

[15] Riaz AA, Ginimol M, Rasha R, Ahmed K, Ahmed A, Catrin S. Transparency in the reporting of artificial intelligence – the TITAN guideline. Premier J Sci. 2025;2. doi: 10.70389/PJS.100082 100082

[16] Poluzzi E, Raschi E, Piccinni C, De Ponti F. Data mining techniques in pharmacovigilance: analysis of the publicly accessible FDA adverse event reporting system (AERS). In: Adem K, editor. Data mining applications in engineering and medicine. Rijeka: InTechOpen; 2012. Ch. 12 p. doi: 10.5772/50095

[17] Lu L, Tian L. Postmenopausal osteoporosis coexisting with sarcopenia: the role and mechanisms of estrogen. J Endocrinol. 2023;259(1):e230116. doi: 10.1530/JOE-23-0116 37523234

[18] Väänänen HK, Härkönen PL. Estrogen and bone metabolism. Maturitas. 1996;23 Suppl:S65-9. doi: 10.1016/0378-5122(96)01015-8 8865143

[19] Wardlaw GM. Putting body weight and osteoporosis into perspective. Am J Clin Nutr. 1996;63(3 Suppl):433S-436S. doi: 10.1093/ajcn/63.3.433 8615336

[20] Wildner M, Peters A, Raghuvanshi VS, Hohnloser J, Siebert U. Superiority of age and weight as variables in predicting osteoporosis in postmenopausal white women. Osteoporos Int. 2003;14(11):950–6. doi: 10.1007/s00198-003-1487-z 13680102

[21] LeBoff MS, Greenspan SL, Insogna KL, Lewiecki EM, Saag KG, Singer AJ. The clinician’s guide to prevention and treatment of osteoporosis. Osteoporos Int. 2022;33(10):2049–102. doi: 10.1007/s00198-021-05900-y 35478046 PMC9546973

[22] Wang Y, Wang Q, Xu Q, Li J, Zhao F. Single-cell RNA sequencing analysis dissected the osteo-immunology microenvironment and revealed key regulators in osteoporosis. Int Immunopharmacol. 2022;113(Pt A):109302. doi: 10.1016/j.intimp.2022.109302 36257255

[23] Wang L-T, Chen L-R, Chen K-H. Hormone-related and drug-induced osteoporosis: a cellular and molecular overview. Int J Mol Sci. 2023;24(6):5814. doi: 10.3390/ijms24065814 36982891 PMC10054048

[24] Fournier MR, Targownik LE, Leslie WD. Proton pump inhibitors, osteoporosis, and osteoporosis-related fractures. Maturitas. 2009;64(1):9–13. doi: 10.1016/j.maturitas.2009.07.006 19674854

[25] Mu Y, Zhou Y, Zhang X, Shao Y. Exploring the mechanisms and targets of proton pump inhibitors-induced osteoporosis through network toxicology, molecular docking, and molecular dynamics simulations. Front Pharmacol. 2025;16:1592048. doi: 10.3389/fphar.2025.1592048 40421218 PMC12104171

[26] Shanika LGT, Reynolds A, Pattison S, Braund R. Proton pump inhibitor use: systematic review of global trends and practices. Eur J Clin Pharmacol. 2023;79(9):1159–72. doi: 10.1007/s00228-023-03534-z 37420019 PMC10427555

[27] Sardar A, Verma S, Raj A, Maji B, Trivedi R. Comparison of differences in cell migration during the osteogenic and adipogenic differentiation of the bone marrow-derived stem cells. J Bone Metab. 2025;32(2):69–82. doi: 10.11005/jbm.25.841 40537102 PMC12183369

[28] Bai J, Ge G, Wang Q, Li W, Zheng K, Xu Y, et al. Engineering stem cell recruitment and osteoinduction via bioadhesive molecular mimics to improve osteoporotic bone-implant integration. Research (Wash). 2022;2022:9823784. doi: 10.34133/2022/9823784 36157511 PMC9484833

[29] Liu H, Song P, Zhang H, Zhou F, Ji N, Wang M, et al. Synthetic biology-based bacterial extracellular vesicles displaying BMP-2 and CXCR4 to ameliorate osteoporosis. J Extracell Vesicles. 2024;13(4):e12429. doi: 10.1002/jev2.12429 38576241 PMC10995478

[30] Anginot A, Nguyen J, Abou Nader Z, Rondeau V, Bonaud A, Kalogeraki M, et al. WHIM syndrome-linked CXCR4 mutations drive osteoporosis. Nat Commun. 2023;14(1):2058. doi: 10.1038/s41467-023-37791-4 37045841 PMC10097661

[31] Fresno Vara JA, Casado E, de Castro J, Cejas P, Belda-Iniesta C, González-Barón M. PI3K/Akt signalling pathway and cancer. Cancer Treat Rev. 2004;30(2):193–204. doi: 10.1016/j.ctrv.2003.07.007 15023437

[32] Diao H, Yang H, Yu B, Fan Y, Li S, Fan J, et al. 5,7-Dihydroxy-4-Methylcoumarin enhances osteogenesis and ameliorates osteoporosis via the AKT1 pathway. Biochem Pharmacol. 2025;233:116752. doi: 10.1016/j.bcp.2025.116752 39800268

[33] Luo H, Zhang J, Zhang F, Peng W. SRSF1 and SRSF2 synergistically regulate Bim expression to mediate glucocorticoid-induced apoptosis in osteoblasts. Biochem Biophys Res Commun. 2025;770:152015. doi: 10.1016/j.bbrc.2025.152015 40381239

[34] You L, Gu W, Chen L, Pan L, Chen J, Peng Y. MiR-378 overexpression attenuates high glucose-suppressed osteogenic differentiation through targeting CASP3 and activating PI3K/Akt signaling pathway. Int J Clin Exp Pathol. 2014;7(10):7249–61. 25400823 PMC4230144

[35] Sims NA, Gooi JH. Bone remodeling: multiple cellular interactions required for coupling of bone formation and resorption. Semin Cell Dev Biol. 2008;19(5):444–51. doi: 10.1016/j.semcdb.2008.07.016 18718546

[36] Chotiyarnwong P, McCloskey EV. Pathogenesis of glucocorticoid-induced osteoporosis and options for treatment. Nat Rev Endocrinol. 2020;16(8):437–47. doi: 10.1038/s41574-020-0341-0 32286516

[37] Nickolas TL, Yin MT, Hong T, Mugwanya KK, Branch AD, Heffron R, et al. Impact of Tenofovir-based pre-exposure prophylaxis on biomarkers of bone formation, bone resorption, and bone mineral metabolism in HIV-negative adults. Open Forum Infect Dis. 2019;6(10):ofz338. doi: 10.1093/ofid/ofz338 31660332 PMC6778426

[38] Liu Z-W, Zhang B-B, Kwok KW-H, Dong X-L, Wong K-H. Network pharmacology analysis and biological validation systemically identified the active ingredients and molecular targets of Kudzu root on osteoporosis. Int J Mol Sci. 2025;26(3):1202. doi: 10.3390/ijms26031202 39940967 PMC11818621

