# Supplementary material for: FAERS-based pharmacovigilance and network toxicology analysis of esomeprazole-associated osteoporosis
Source: PLoS One. 2026 Aug 4;21(8):e0353264. doi: 10.1371/journal.pone.0353264 (PMC13436734; doi:10.1371/journal.pone.0353264)
Supplement: Supplementary file 1 [file pone.0353264.s001.docx]

supplementary materialsa

S1 Table: 2x2 Table for Disproportionality Analysis

| Item | Target adverse events reported | Other adverse events reported | Total |
| --- | --- | --- | --- |
| Target drugs | a | b | a + b |
| Other drugs | c | d | c + d |
| Total | a + c | b + d | a + b + c + d |
