# Supplementary material for: FAERS-based pharmacovigilance and network toxicology analysis of esomeprazole-associated osteoporosis
Source: PLoS One. 2026 Aug 4;21(8):e0353264. doi: 10.1371/journal.pone.0353264 (PMC13436734; doi:10.1371/journal.pone.0353264)
Supplement: Supplementary file 2 [file pone.0353264.s002.docx]

supplementary materialsa

S2 Table: Signal Detection Methods, Formulas, and Generation Criteria

| Method | Calculation formula | Criteria |
| --- | --- | --- |
| ROR | $\text{ROR=}\frac{\text{a / c}}{\text{b}\text{ / }\text{d}}$ | a ≥ 3  95%CI (lower limit) > 1 |
|  | $\text{SE}\text{(}\text{lnROR}\text{)=}\sqrt{\frac{\text{1}}{\text{a}}\text{+}\frac{\text{1}}{\text{b}}\text{+}\frac{\text{1}}{\text{c}}\text{+}\frac{\text{1}}{\text{d}}}$ |  |
|  | $\text{95\%}\text{CI}\text{= }\text{e}^{\ln\left( \text{ROR} \right)\text{±1.96}\text{se}}$ |  |
| PRR | $\text{PRR=}\frac{\text{a / (a+b)}}{\text{c}\text{ / (}\text{c}\text{+}\text{d}\text{)}}$ | a ≥ 3  PRR ≥ 2  95%CI (lower limit) > 1 |
|  | $\text{SE}\text{(}\text{lnPRR}\text{)=}\sqrt{\frac{\text{1}}{\text{a}}\text{−}\frac{\text{1}}{\text{a+b}}\text{+}\frac{\text{1}}{\text{c}}\text{−}\frac{\text{1}}{\text{c+d}}}$ |  |
|  | $\text{95\%}\text{CI}\text{= }\text{e}^{\ln\left( \text{PRR} \right)\text{±1.96}\text{se}}$ |  |
|  | $\text{χ2 =}\frac{\text{ }\text{(ad−bc)}^{\text{2}}\text{(a+b+c+d)}}{\text{( a+b)(a+c)(c+d)(b+d)}}$ | a ≥ 3  PRR ≥ 2  $\text{χ2}\text{≥4}$ |
| BCPNN | IC=$\text{log}_{\text{2}}\frac{\text{p}\text{(}\text{x}\text{,}\text{y}\text{)}}{\text{p}\text{(}\text{x}\text{)}\text{p}\text{(}\text{y}\text{)}}\text{=}\text{log}_{\text{2}}\frac{\text{a}\text{(}\text{a}\text{+}\text{b}\text{+}\text{c}\text{+}\text{d}\text{)}}{\text{(}\text{a}\text{+}\text{b}\text{)(}\text{a}\text{+}\text{c}\text{)}}$ | IC025>0 |
|  | E(IC)=$\text{log}_{\text{2}}\frac{\text{(}\text{a}\text{+}\text{γ}\text{11)(}\text{a}\text{+}\text{b}\text{+}\text{c}\text{+}\text{d}\text{+}\text{α}\text{)(}\text{a}\text{+}\text{b}\text{+}\text{c}\text{+}\text{d}\text{+}\text{β}\text{)}}{\text{（}\text{a}\text{+}\text{b}\text{+}\text{c}\text{+}\text{d}\text{+}\text{γ}\text{）(}\text{a}\text{+}\text{b}\text{+}\text{α}\text{1)(}\text{a}\text{+}\text{c}\text{+}\text{β}\text{1)}}$ |  |
|  | $\text{V}\left( \text{IC} \right)\text{=}\frac{\text{1}}{{\text{(}\text{ln}\text{2)}}^{\text{2}}}\text{\{}\left[ \frac{\left( \text{a}\text{+}\text{b}\text{+}\text{c}\text{+}\text{d} \right)\text{−}\text{a}\text{+}\text{γ}\text{−}\text{γ}\text{11}}{\left( \text{a}\text{+}\text{γ}\text{11} \right)\left( \text{1+}\text{a}\text{+}\text{b}\text{+}\text{c}\text{+}\text{d}\text{+}\text{γ} \right)} \right]\text{+}\left[ \frac{\left( \text{a}\text{+}\text{b}\text{+}\text{c}\text{+}\text{d} \right)\text{−}\left( \text{a}\text{+}\text{b} \right)\text{+}\text{α}\text{−}\text{α}\text{1}}{\left( \text{a}\text{+}\text{b}\text{+}\text{α}\text{1} \right)\left( \text{1+}\text{a}\text{+}\text{b}\text{+}\text{c}\text{+}\text{d}\text{+}\text{α} \right)} \right]\text{+}\left[ \frac{\left( \text{a}\text{+}\text{b}\text{+}\text{c}\text{+}\text{d} \right)\text{−}\left( \text{a}\text{+}\text{c} \right)\text{+}\text{β}\text{−}\text{β}\text{1}}{\left( \text{a}\text{+}\text{c}\text{+}\text{β}\text{1} \right)\left( \text{1+}\text{a}\text{+}\text{b}\text{+}\text{c}\text{+}\text{d}\text{+}\text{β} \right)} \right]\text{\}}$ |  |
|  | $\text{γ}\text{=}\text{γ}\text{11}\frac{\text{(}\text{a}\text{+}\text{b}\text{+}\text{c}\text{+}\text{d}\text{+}\text{α}\text{)(}\text{a}\text{+}\text{b}\text{+}\text{c}\text{+}\text{d}\text{+}\text{β}\text{)}}{\text{(}\text{a}\text{+}\text{b}\text{+}\text{α}\text{1)(}\text{a}\text{+}\text{c}\text{+}\text{β}\text{1)}}$ |  |
|  | *IC-2SD=E(IC)-2*$\sqrt{\text{V}\text{(}\text{IC}\text{)}}$  $\text{α}\text{1=}\text{β}\text{1=1；}\text{α}\text{=}\text{β}\text{=2；}\text{γ}\text{11=1}$ |  |
| EBGM | $\text{EBGM=}\frac{\text{a}\text{(}\text{a}\text{+}\text{b}\text{+}\text{c}\text{+}\text{d}\text{)}}{\left( \text{a}\text{+}\text{c} \right)\text{(}\text{a}\text{+}\text{b}\text{)}}$ | EBGM05>2 |
|  | $\text{SE}\text{(}\text{lnEBGM}\text{)=}\sqrt{\frac{\text{1}}{\text{a}}\text{+}\frac{\text{1}}{\text{b}}\text{+}\frac{\text{1}}{\text{c}}\text{+}\frac{\text{1}}{\text{d}}}$ |  |
|  | $\text{95\%}\text{CI}\text{= }\text{e}^{\ln\left( \text{EBGM} \right)\text{±1.96}\text{se}}$ |  |
