# Supplementary material for: FAERS-based pharmacovigilance and network toxicology analysis of esomeprazole-associated osteoporosis
Source: PLoS One. 2026 Aug 4;21(8):e0353264. doi: 10.1371/journal.pone.0353264 (PMC13436734; doi:10.1371/journal.pone.0353264)
Supplement: Supplementary file 3 [file pone.0353264.s003.docx]

supplementary materialsa

S3 Table: AutoDock Vina docking parameters for esomeprazole with core target proteins

| **Target** | **Center x** | **Center y** | **Center z** | **Size x** | **Size y** | **Size z** | **Energy range** | **Num modes** | **Exhaustiveness** |
| --- | --- | --- | --- | --- | --- | --- | --- | --- | --- |
| CXCR4 | 3.591 | 6.89 | 40.116 | 56 | 88 | 78 | 5 | 20 | 8 |
| AKT1 | -27.253 | -21.535 | 19.46 | 58 | 112 | 78 | 5 | 20 | 8 |
| CASP3 | -99.155 | 30.446 | 67.441 | 56 | 60 | 60 | 5 | 20 | 8 |
