# Supplementary material for: FAERS-based pharmacovigilance and network toxicology analysis of esomeprazole-associated osteoporosis
Source: PLoS One. 2026 Aug 4;21(8):e0353264. doi: 10.1371/journal.pone.0353264 (PMC13436734; doi:10.1371/journal.pone.0353264)
Supplement: Supplementary file 4 [file pone.0353264.s004.docx]

supplementary materialsa

S4 Table: Chemical Information of Esomeprazole

| Plasticizer | Chemical Formula | SMILES Structure | MW (g/mol) |
| --- | --- | --- | --- |
| Esomeprazole | C17H18N3O3S | CC1=CN=C(C(=C1OC)C)C[S@](=O)C2=NC3=C(N2)C=C(C=C3)OC | 345.42 |
